# Commentary: Effect of Probiotic Supplementation on Cognitive Function and Metabolic Status in Alzheimer's Disease: A Randomized, Double-Blind and Controlled Trial

**DOI:** 10.3389/fnagi.2018.00054

**Published:** 2018-03-06

**Authors:** Friedrich Leblhuber, Michael Egger, Burkhard Schuetz, Dietmar Fuchs

**Affiliations:** ^1^Department of Gerontology, Johannes Kepler University of Linz, Linz, Austria; ^2^Biovis Diagnostik MVZ GmbH, Limburg, Germany; ^3^Division of Biological Chemistry, Biocenter, Innsbruck Medical University, Innsbruck, Austria

**Keywords:** intestinal microbiota, brain-gut-axis, neuroinflammation, cognitive decline, tryptophan metabolism

Akbari and colleagues (Front. Aging Neurosci. 8:256. doi: 10.3389/fnagi.2016.00256) in their randomized, double blind clinical trial demonstrate that probiotic administration for 12 weeks had favorable cognitive and metabolic effects in their patients with Alzheimer's disease (AD). Most impressive was the significant improvement in the mini mental state examination [MMSE (*p* < 0.001)]. Some metabolic parameters such as plasma malondialdehyde, markers of insulin metabolism and serum triglycerides were also significantly different in the AD patients compared with the control group, and probably caused by probiotic treatment. Additionally, serum high sensitive C-reactive protein (hs-CRP) significantly changed after probiotic supplementation (*p* < 0.001). However, changes in other biomarkers of oxidative stress and inflammation were negligible. The authors concluded that evaluation of other biomarkers of inflammation and oxidative stress would be informative.

Concerning this suggestion several different fecal and serum inflammation markers in correlation to intestinal bacterial strains were investigated in our recent study on the role of gut microbiota in patients with cognitive decline (Leblhuber et al., 2017). From a subgroup of 23 patients (9 females, 14 males, aged 78 ± 8.5 years) out of 55 consecutive outpatients with symptoms of cognitive decline, intestinal bacterial taxa and immune system as well as inflammation biomarkers in serum and stool specimens were investigated.

Confirming our earlier findings (Widner et al., 2000; Wissmann et al., 2013; Leblhuber et al., 2015), signs of immune activation could be detected: serum neopterin was found elevated as well as the Kyn/Trp ratio, an index of tryptophan breakdown by enzyme indoleamine 2,3-dioxygenase-1 (IDO). Most interestingly, a close correlation was found between fecal S100A12 and serum neopterin (*p* < 0.001, see Figure 1), indicating coincident low grade systemic and intestinal inflammation (Caracciolo et al., 2014). There was no influence of gender. These findings again underline the role of gut inflammation as a possible pathogenic cofactor in cognitive deterioration and dementia.

**Figure 1:**
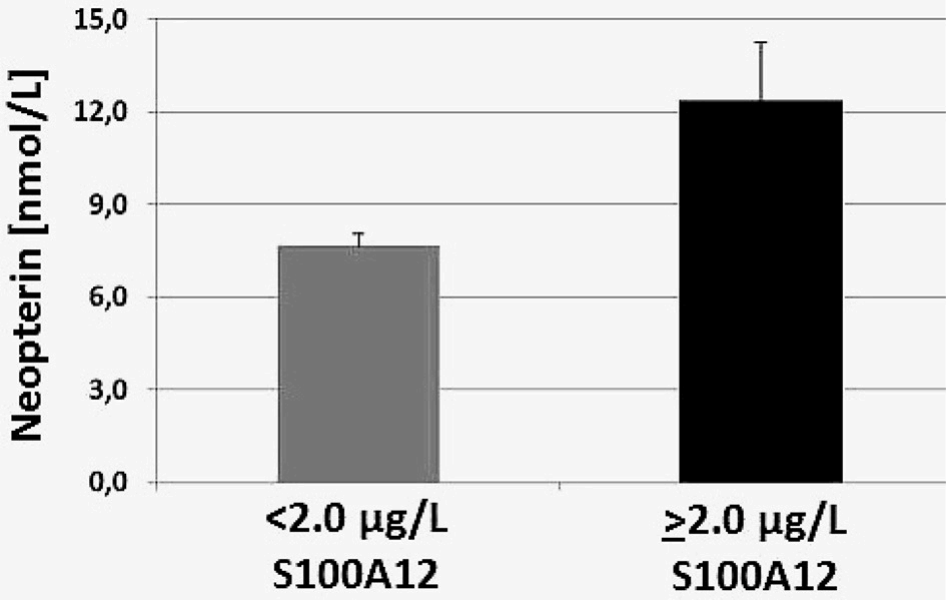
Serum neopterin concentration (mean ± SEM) in patients suffering from cognitive decline grouped according to fecal S100A12 concentrations (*p* < 0.001), modified from Leblhuber et al. (2017).

In an earlier study (Shepherd et al., 2006), the potential role of pro-inflammatory S100A9 and S100A12 proteins in the pathogenesis of AD was described. Circulating CRP, known to affect cognition negatively, was elevated in our series (1.6 ± 2.3 mg/L) without clinical signs of acute infection as in the study of Akbari et al. but further indicating low grade inflammation (“inflammaging”) (Caracciolo et al., 2014) in this group of patients. We found pro-inflammatory *Clostridium Cluster I* significantly correlated with anti-inflammatory *Faecalibacterium prausnitzii* (*p* < 0.01).

In a recent paper (Cattaneo et al., 2017) the stool abundance of selected bacterial stool taxa including *F. prausnitzii* and the blood levels of pro- and anti-inflammatory cytokines in cognitively impaired patients and in a group of controls was measured. Amyloid positive patients showed higher levels of pro-inflammatory cytokines compared with both controls and with amyloid negative patients. A possible causal relation between gut microbiota related inflammation and amyloidosis was suspected in this study.

In our series *F. prausnitzii* correlated with MMSE (*p* < 0.05), with *Akkermansia muciniphila* (*p* < 0.01) and with serum neopterin (*p* < 0.05). Further, a strong correlation was found between anti-inflammatory α1-antitrypsin and pro-inflammatory S100A12 (*p* < 0.001) in the fecal specimens of our cognitively impaired patients. The anti-inflammatory action of α1-antiptrypsin on microglial mediated neuroinflammation could be shown *in vitro* (Gold et al., 2014). In our series, α1-antitrypsin was also correlated with zonulin (*p* < 0.01), a protein modulating tight junction permeability between cells of the digestive tract. All these findings together may indicate changes in the microbiota-gut-brain-axis correlated to neuroinflammation during cognitive decline. Because neuroinflammation is an early event in the pathogenesis of dementia (Caracciolo et al., 2014) these markers may be important in the very beginning of this devastating process.

The increased immune activation and inflammation in AD could indeed relate to the age-related changes of gut microbiota as is indicated by the close relationship between fecal S100A12 and serum neopterin concentrations (Leblhuber et al., 2017). Unfortunately, in the above mentioned study (Akbari et al., 2016) these and additional inflammation markers were not measured except hs-CRP; as the authors stated the measurement of fecal bacteria loads before and after probiotic supplementation was “very difficult” in their study.

Overall, the role of probiotics in preventing dementia seems promising and should be further elucidated in future studies. These investigations should include the bacterial taxa as well as the serum and intestinal inflammation markers mentioned above together with the metabolic parameters mentioned by Akbari et al. (2016). A possible personalized therapy for cognitive decline and dementia as well as for establishing effective nutritional interventions with pre- and probiotics for healthy brain aging should be considered (Zamroziewicz and Barbey, 2016).

## Author contributions

All authors have made substantial contribution and critical revision to this manuscript.

### Conflict of interest statement

The authors declare that the research was conducted in the absence of any commercial or financial relationships that could be construed as a potential conflict of interest.
